# Relation of Hygiene to Practical Dentistry

**Published:** 1885-11

**Authors:** C. E. H. Phillips

**Affiliations:** New York


### RELATION OF HYGIENE TO PRACTICAL DENTISTRY.

BY C. E. H. PHILLIPS, D. D. S., NEW YORK.

The great advance made in recent years in treating diseases of the mouth is certainly encouraging, and though excellent results are by no means uncommon, we should not rest here. Patients must be so instructed as to aid us in our endeavors, and not until they are impressed with the importance of the hygienic care of the mouth is our duty fully performed. The unwarrantable neglect of many patients in this regard is astonishing.

The physician, who by careful treatment and strict enforcement of sanitary laws has stamped out from a family or community the scourge of malignant fever, with precautionary instructions as to ventilation, diet and hygiene, for the protection of its other members, would at least feel discouraged, and justly so, should a recurrence of the disease result from gross neglect of sanitary conditions, or carelessness as to instructions given.

How very often is the dental practitioner subjected to like discouragement. Having corrected diseased conditions in the oral cavity, carefully cut out all carious dentine and nicely filled the cavities, removed accumulated calculous and mucous stains from the teeth and polished their surfaces, resulting not only in a pleasing appearance, but to the operator the consciousness of having performed saving work and aided the general health, and after faithful instruction as to thorough and careful use of the brush, with a good dentifrice, at proper periods, the employment of waxed floss silk and quill toothpick, is it to be wondered at if a feeling very akin to disgust is experienced, if in a few months or a year the patient again presents himself with tartar already forming in quantities, the teeth yellow from mucous attachment, the gums soft and spongy, ready to ooze blood from their gingival edges upon the slightest touch, all evidencing little care or use of the brush, or, possibly, its total disuse and general disregard to common hygienic requirements?

There is no question that the great essential to a healthy mouth, wholesome saliva, and pure breath, is *cleanliness.* Not until the dentist is able to force upon his patients the necessity of *thorough* cleanliness of the mouth at all times, and has their assistance to this end, shall we save teeth and keep the surrounding tissues in a state of health, and not until then should we rest satisfied.

